# Implementation of the ID-PALL Assessment Tool for Palliative Care Needs: A Feasibility and Prevalence Study in a Tertiary Hospital

**DOI:** 10.1089/pmr.2023.0080

**Published:** 2024-08-05

**Authors:** F. Teike Lüthi, M. Bernard, G. Behaghel, S. Burgniard, P. Larkin, G. D. Borasio

**Affiliations:** ^1^ Palliative and Supportive Care Service, Lausanne University Hospital and University of Lausanne, Lausanne, Switzerland.; ^2^ Chair of Palliative Care Nursing, Palliative and Supportive Care Service, Lausanne University Hospital and University of Lausanne, Lausanne, Switzerland.

**Keywords:** feasibility, hospital, ID-PALL, nurses, palliative care, physicians

## Abstract

**Background:**

Identifying patients who require palliative care is a major public health concern. ID-PALL is the first screening instrument developed and validated to differentiate between patients in need of general versus specialized palliative care.

**Objectives:**

This study aimed to (1) evaluate user satisfaction and the facilitators and barriers for ID-PALL use and (2) assess the prevalence of patients who require palliative care.

**Design:**

A mixed methods study with an explanatory sequential design.

**Setting/Subjects:**

Over a six-month period, patients admitted to two internal medicine wards of a Swiss tertiary hospital were screened by nurses and physicians with ID-PALL, two to three days after hospitalization. Nurses and physicians completed a questionnaire and participated in focus groups.

**Results:**

Out of 969 patients, ID-PALL was completed for 420 (43.3%). Sixty percent of patients assessed needed general palliative care and 26.7% specialized palliative care. From the questionnaire and focus groups, five subthemes were identified concerning facilitators and barriers: organization, knowledge, collaboration, meaning, and characteristics of the instrument. ID-PALL was recognized as an easy-to-use and helpful instrument that facilitates discussion between health care professionals about palliative care. The difficulties in using ID-PALL in nurse–physician collaboration and the paucity of referrals to the palliative care team were highlighted.

**Conclusions:**

ID-PALL helped to identify a very high prevalence of palliative care needs among internal medicine patients in a tertiary hospital setting. Although regarded as helpful and easy to use, challenges remain concerning interprofessional implementation and inclusion of palliative care specialists, which may be met by automatic referrals in case of specialist needs.

## Introduction

The main goal of palliative care is to improve the quality of life of patients faced with life-threatening illnesses and their relatives.^1–3^ Depending on care settings and pathologies, the prevalence of patients with potential palliative care needs can be as high as 82%.^4–10^ Evidence suggests that palliative care interventions improve symptom management, patient satisfaction, patients’ and relatives’ quality of life, and the opportunity to die at home.^11–16^ An important barrier to achieving these goals is the difficulty health care professionals face in identifying patients in need of palliative care.^17–27^ International recommendations emphasize the necessity for standardized identification processes based not only on predicting mortality but also on anticipating palliative care needs, to ensure equitable access to palliative care.^8,28–30^

### The ID-PALL instrument

ID-PALL (IDentification of patients in need of PALLiative care) is a two-step instrument that, to the best of our knowledge, is the first validated screening instrument to differentiate between patients in need of general versus specialized palliative care^31–33^ (Supplementary Data S1). It can be used by nurses and physicians alike. The first part (seven items) is designed to identify patients in need of general palliative care (ID-PALL G), whereas the second part (eight items) aims to identify patients requiring specialized palliative care (ID-PALL S). General palliative care refers to individuals facing a life-threatening, progressive, incurable illness, or who have reached the terminal phase of their lives but do not present complex issues and should not usually require the involvement of palliative care specialists. Approximately 80% of patients in need of palliative care require general palliative care.^33^ The remaining 20% of patients present complex issues, including unstable clinical conditions, treatment-refractory symptoms, and/or high levels of existential suffering, necessitating an intervention by palliative care specialists. ID-PALL S is assessed only if ID-PALL G is positive. Each item is evaluated using a “yes/no” response format. A single positive response to any item in either part is considered the cutoff point for determining the need for either general or specialized palliative care.^32^

To improve the clinicians’ experience with ID-PALL, we supplemented the instrument with ten clinical recommendations for the management of ID-PALL-G patients (Supplementary Data S1; for details see Refs. 31 and 32).

### Study objectives

The main objective of this study was to evaluate the facilitators and barriers to ID-PALL use, as well as user satisfaction. A second objective was to assess the prevalence of patients requiring palliative care in the setting studied.

## Materials and Methods

### Study design

Mixed method study based on an explanatory sequential design.^34^

### Setting

This study was conducted in two internal medicine wards of a tertiary hospital in the French linguistic region of Switzerland.

#### Feasibility and acceptability

A questionnaire was created based on implementation science recommendations,^35^ assessing acceptability, adoption, appropriateness, feasibility, expansion (the possibility to implement ID-PALL in other services), implementation barriers and facilitators, coverage (changes in clinical practice with ID-PALL), and sustainability (intention to continue to use ID-PALL) with a 4-point Likert scale (“strongly disagree,” “disagree,” “agree,” and “strongly agree”). Responses were dichotomized into two categories: “agree” and “not agree”. Fourteen questions assessed the feasibility of using ID-PALL and seven the clinical recommendations.

Focus groups (minimum three participants)^36^ were held by the researchers (F.T.L., M.B., or G.B) and digitally recorded with permission. Each focus group was led by one researcher with another assisting in taking notes. The participants completed a demographic questionnaire. The interview guide comprised three parts: (1) global knowledge of palliative care, (2) use of ID-PALL, and (3) use of clinical recommendations.

#### Prevalence of patients who require palliative care

##### Procedure of ID-PALL completion

ID-PALL was made available to clinical staff as an electronic form, together with the clinical recommendations. To strengthen the likelihood of completion, ID-PALL was reviewed by clinical leads in nursing and medicine to ensure that filling out the new instrument would not cause an additional workload for teams and could be incorporated into the daily clinical routine. At the beginning of the study, a one-hour teaching session on palliative care and ID-PALL was given to the nurses and physicians involved. The nurses were primarily responsible for completing ID-PALL, if possible, together with the physicians (e.g., during clinical rounds). If not, the nurses completed ID-PALL and discussed it afterward with the physicians, including the clinical strategy (e.g., whether to call the palliative care team). Patients were screened by nurses and physicians for eligibility after two days of hospitalization (this period could be extended to three days if a patient arrived in the unit during the weekend) if they were over 18 years of age and were hospitalized in one of two participating internal medicine wards. The timeframe of the study was extended from the planned four to six months (August 2020–February 2021) owing to the COVID-19 pandemic, which caused a reduction in ID-PALL completion rates because of the high patient turnover. The questionnaires were administered at the end of February, and the focus groups took place between March and April 2021.

### Data collection

A questionnaire was sent to all nurses and physicians working in these units at the end of data collection, and all were invited to participate in the focus groups.

### Data analyses

Descriptive statistics were reported as frequencies and percentages for categorical variables and as medians and standard deviations for continuous variables. Chi-square tests were used to analyze the categorical differences between the groups. Independent *t* tests were used to examine significant associations between the independent means. The significance level was set at *p* < 0.05 for all tests. Statistical analyses were performed using IBM SPSS Statistics 27.

Regarding the focus groups, notes were documented, and audio recordings were transcribed and checked for accuracy by F.T.L. and G.B. Thematic analysis was guided by Braun and Clarke’s method.^37,38^ A deductive approach was chosen based on the facilitators and barriers to the implementation of an instrument, followed by an inductive approach to identify subthemes. Initially, 10% of the data was parallel blind coded (F.T.L. and G.B.). After comparing subthemes and discussing and resolving dissents, an initial codebook was developed (F.T.L.) that was applied to the remaining data. Codes were developed into themes and subthemes (F.T.L. and G.B.) and discussed with M.B. for agreement.

This study was approved on July 21, 2020, by the institutional ethics committee (n° 2020-11).

## Results

### Feasibility and acceptability

In both units, 36 of 42 nurses (86%) and 5 of 8 physicians (63%) responded to the questionnaire. One nurse returned the questionnaire without having used ID-PALL and was excluded. Demographic characteristics are shown in Table 1. Thirty-three participants were women (80%). Only three nurses (8%) had received specific training in palliative care: one received a three-day training and two received a 20-day general palliative care training course.

**Table 1.** Professional and Demographic Characteristics of the Respondents

Characteristics	Total sample *n* = 40 *N* (%)
Profession	
Nurses	35 (88%)
Physicians	5 (12%)
Gender	
Women	32 (80%)
Men	8 (20%)
Practice since diploma	
<1 year	5 (13%)
1–5 years	19 (47%)
6−10 years	6 (15%)
>10 years	10 (25%)
Practice in the medicine ward	
<1 year	5 (12%)
1–5 years	21 (53%)
6−10 years	7 (18%)
>10 years	6 (15%)
No answer	1 (2%)
Training in palliative care	
Yes	4 (10%)
No	36 (90%)
Wish for a specific training	
Yes	25 (63%)
No	10 (25%)
No answer	5 (12%)

Out of the 40 professionals who responded to the questionnaire, 36 found that using ID-PALL was compatible with their daily professional activity (90%), 37 found it easy to complete (93%), especially in the electronic medical file, 35 perceived it as useful for identifying palliative care needs (88%), and 39 thought it could be used in other services (98%). ID-PALL was seen as facilitating consultation with the palliative care team by 28 professionals (70%), useful for discharge orientation by 27 (68%), and could be recommended to colleagues by 27 (68%). Twenty-five participants identified a positive impact of ID-PALL on patients (63%), 21 on health care professionals (55%), and 19 on relatives (48%). Only 13 professionals reported having changed their practice because of the use of ID-PALL (33%) and 29 wished to continue to use ID-PALL after the study (63%).

While 25 participants (65%) were aware of the clinical recommendations, only 14 nurses (35%) consulted them. Thirteen found the recommendations clear (93%), 11 found them relevant to clinical practice (79%), and 10 wanted training to ensure that they were applying recommendations correctly (71%).

#### Focus groups

Four focus groups were conducted, two with three nurses (*n* = 6) and two with three physicians (*n* = 6), each lasting 30–60 minutes. All nurses and three of the physicians were women. Three nurses had less than three years of clinical experience, and the others had more than seven years. Five physicians were residents and one was a senior registrar, all of whom had less than five years of experience. Only one nurse had a three-day training in palliative care.

The results were divided into two main categories: facilitators and barriers, both subdivided into subthemes: organization, knowledge (only in barriers), collaboration, meaning, and characteristics of the instrument.

#### Facilitators

##### Organization

The integration of ID-PALL into the electronic medical file facilitated its use and access to the score. Good knowledge of the patient facilitated the use of ID-PALL. Nurses decided to make ID-PALL completion mandatory in the nursing documents after 48 hours of hospitalization to facilitate the systematization of the screening.

##### Collaboration

The use of ID-PALL was recognized as an opportunity to open interdisciplinary discussions between nurses and physicians about patients and palliative care. For the majority, it made sense to complete the instrument together which was sometimes helpful for modifying the therapeutic plan. Nurses felt capable and legitimized to complete ID-PALL within the interprofessional team, even if they sometimes struggled with their advocacy role.

##### Meaning

The first meaning attributed to ID-PALL was to support the rationale for contacting palliative care specialists. In addition, four participants explained that ID-PALL was used to confirm their prior subjective clinical judgment that the patient required specialist palliative care, thus providing an objective assessment of their clinical impression. Participants also noted that ID-PALL helped them to address unclear clinical situations as a way of thinking more broadly about palliative care for patients and rendered them more aware of general palliative care and their role in it.

##### Characteristics of ID-PALL

The format of the instrument was appreciated particularly the binary responses. Most participants described ID-PALL as easy to use with clear and simple items. Participants felt that ID-PALL was useful for discussing palliative care with colleagues and helpful for inexperienced professionals.

#### Barriers

##### Organization

Participants said that using ID-PALL made them ask new questions about the patient’s clinical situation (e.g., could he die in the next 12 months? What does he really know about his situation? How is the family coping?). This, in turn, increased the length of exchange between the nurse and the doctor and thus their workload. Although recognized as valuable, ID-PALL was seen by some as difficult to apply in daily practice. Finding the appropriate time for the interprofessional completion of ID-PALL was a challenge. It was sometimes difficult to complete ID-PALL in the first 48 hours after admission because professionals did not know the patients well enough.

##### Knowledge

None of the participants read the ID-PALL instructions for use. The need for palliative care training, including the skills required to initiate a discussion on palliative care for patients, was highlighted. Negative connotations of palliative care by both patients and professionals were stressed as barriers to completing ID-PALL, which was mainly seen as an instrument facilitating referral to palliative care specialists. Most participants were unaware of the clinical recommendations provided with the instrument.

##### Collaboration

Nurses noted the lack of interest and availability of some physicians to complete ID-PALL and more generally to address the issue of palliative care. To be solely responsible for completing ID-PALL was difficult for nurses, who felt that they were pestering physicians to discuss ID-PALL and palliative care. When physicians frequently decided not to refer to palliative care specialists despite a positive ID-PALL S, it created moral distress for the nurses. Most physicians found that completing ID-PALL with nurses took too much time because they felt that the nurses needed to talk about their emotional experiences. Nurses were often seen as “record keepers,” filling in ID-PALL according to what the doctors asked them to tick. Physicians never completed ID-PALL on their own. Nurses hoped that using ID-PALL would enable patients to make earlier referrals to palliative care as nurses often requested it without being heard by physicians. Two physicians suggested automatic referral to palliative care when ID-PALL S is positive.

##### Meaning

Some participants found it difficult to give meaning to the use of ID-PALL. Some completed it out of obligation, others to confirm their previous clinical impressions. Despite the presence of written recommendations, all nurses emphasized the need to receive support concerning the care to be delivered once the patient is ID-PALL G positive. For example, they asked for help on how to use the Edmonton Symptom Assessment System, how to discuss palliative care issues with patients, or how to assess relatives’ needs.

##### Characteristics of ID-PALL

One physician felt that ID-PALL items did not add anything new, except for confirming what he already knew. Another said that there were too many items. One suggested having easier items and a “missing information” option in the responses.

### Prevalence of patients who required palliative care

Between August 2020 and February 2021, 969 patients were admitted in the two wards [480 (49.6%) in the first ward and 489 (50.5%) in the second]. Of these, 40.8% were women, the mean age was 68 years [standard deviation (SD) = 16.9], and the mean length of stay was 9.5 days (SD = 9.8) (Table 2). There were no significant differences between the two wards. The most prevalent diagnoses were cancer (27.7%), chronic obstructive pulmonary disease (20.3%), and congestive heart failure (14.8%). COVID-19 was never documented as the first diagnosis, but 166 patients had a secondary diagnosis of COVID-19 (119 in the first ward and 47 in the second) and 51 died. Of these 51, only five were screened ID-PALL G positive and one ID-PALL S positive. Only one specialist palliative care consultation was requested, whereas 14 consultation referrals were made for patients with COVID-19 who had not been screened with ID-PALL.

**Table 2.** Demographic and Medical Characteristics of the Patients

Variables	Total sample *n* = 969
	*N* (%)/mean (SD)
Gender	
Women	395 (40.8%)
Men	574 (59.2%)
Age	68.3 (16.9)
Length of stay	9.5 (9.8)
Primary hospital diagnosis	
Cancer	268 (27.7%)
Dementia	15 (1.5%)
CHF	143 (14.8%)
COPD	197 (20.3%)
Renal diseases	48 (5.0%)
Neurological disorders	14 (1.4%)
Metabolic disorders	62 (6.4%)
Gastrointestinal diseases	73 (7.5%)
Others	149 (15.4%)

CHF, congestive heart failure; COPD, chronic obstructive pulmonary disease; SD, standard deviation.

ID-PALL was completed for 420 patients (43.3%) in the two medical wards (*n* = 183 (43.6%) in the first ward versus *n* = 237 (56.4%) in the second ward, *p* = 0.001), after a mean of 3.8 days (SD = 7.2) of hospitalization. In total, 86.7% of the patients assessed with ID-PALL showed palliative care needs (60.0% general and 26.7% specialized) (Table 3).

**Table 3.** Prevalence of Patients with Palliative Care Needs according to ID-PALL

Variables	Total sample *n* = 420 *N* (%)	Ward 1 *n* = 183	Ward 2 *n* = 237
No need for PC	56 (13.3%)	28 (15.3%)	28 (11.8%)
Need for general PC	252 (60.0%)	102 (55.7%)	150 (63.3%)
Need for specialized PC	112 (26.7%)	53 (29.0%)	59 (24.9%)

PC, palliative care.

### Association between ID-PALL results and palliative care referrals

We analyzed the association between ID-PALL results and the frequency of referrals to the specialized palliative care team (Table 4).

**Table 4.** Association Between ID-PALL Results and Palliative Care Referrals

	Total sample *n* = 420	No need for PC *n* = 56	Need for general PC *n* = 252	Need for specialized PC *n* = 112
	*N* (%)	*N* (%)	*N* (%)	*N* (%)
Referral to palliative care specialists				
Yes	37 (8.8%)	2 (3.6%)	10 (4.0%)	25 (22.3%)
No	383 (91.2%)	54 (96.4%)	242 (96.0%)	87 (77.7%)

PC, palliative care.

Less than a quarter (22.3%) of ID-PALL S-positive patients were referred to the palliative care team. The most frequent reasons for referral were (1) relief of severe or persistent symptoms (59%), (2) support for the assessment of physical symptoms or psycho-socio-spiritual difficulties (56%), and (3) relief of severe psychosocial or existential suffering of the patient (52%).

## Discussion

This mixed-method study reports the preparation phase of an institutional implementation of ID-PALL, a new instrument for the identification of patients in need of general or specialized palliative care.^32^ ID-PALL was recognized as an easy-to-use and helpful instrument for the identification of palliative care needs, which facilitates discussion between health care professionals about palliative care. The “yes-no” response format of ID-PALL was regarded as a positive feature, akin to observations for similar instruments.^39,40^ Although ID-PALL helped clarify unclear situations, it was mainly used to validate the clinicians’ subjective impression that patients needed palliative care.

ID-PALL was completed for less than half of the hospitalized patients. Barriers to its completion included the difficulty to complete ID-PALL in the first 48 hours, the lack of time for joint completion by physicians and nurses, the choice to fill it in only for patients already presumed to need palliative care, and the difficulty of dealing with the issue of palliative care. These strategies can be likened to avoidance patterns because of a lack of knowledge and skills.^17–21^

Patients identified as requiring specialized palliative care were not systematically referred to the palliative care team. Physicians argued that they knew how to manage their patients and that specialists would not be able to handle all the requests. In a feasibility study using the Supportive and Palliative Care Indicators Tool (SPICT), only 7.8% of the general practitioners (GP) invited took part.^41^ In another study, GPs rarely reported using an identification instrument even when struggling with the identification of palliative care needs of nononcological patients.^42^ Physicians’ difficulties in referring patients to palliative care have long been known, especially in cure-oriented tertiary hospitals with a large number of junior physicians,^25,26,43,44^ who often find it difficult to discuss this topic with the patient.^17,26,42,45^ Barriers to referrals are multifactorial, including restriction of palliative care to terminal cancer patients, lack of knowledge in general palliative care, and communication difficulties with specialists.^17,18,20,21,46^

Participants highlighted the difficulties to complete ID-PALL within 48 hours, which is congruent with comments of participants to implementation studies currently underway. The 48-hour period was devised following recommendations for early identification^47–50^ and increasingly shorter hospital stays (five days on average in the study wards). This does not seem to meet the needs of professionals, who require more time to gain a better understanding of the situation of patients and their families. A “don’t know” column could be added to the “yes-no” answers in ID-PALL so that professionals can fill in ID-PALL within 48 hours while identifying the data they should complete with patients and their relatives.

Basic training in palliative care appears to be a prerequisite for effectively implementing ID-PALL, especially the general palliative care recommendations. In this regard, nurses play an important role in supporting patients and their relatives and coaching junior physicians.^19,44^ More undergraduate and postgraduate interprofessional training is urgently required to facilitate nurse–physician interactions and reduce interprofessional practice variations and moral distress mainly on the nurses’ side.^8,19,45,51–54^

The proportion of patients in need of palliative care in our study was high, particularly regarding general palliative care. While other studies do not distinguish between general and specialized palliative care needs, the reported prevalence rates are as high as 82%, which is close to our data.^4–6,8,9^ One likely reason for this is that participants used ID-PALL mainly as an instrument to validate their clinical impression that certain patients needed palliative care, rather than screening all patients. However, this entails the risk of missing out on patients whose palliative care needs are not immediately recognizable.

This implementation study revealed important interprofessional challenges. Nurses, because of their proximity to patients and their advocacy role,^55,56^ often relay the need to call the palliative care team. This is why we decided to give nurses responsibility for filling in ID-PALL.^31,32,57^ However, it is also an explicit aim of ID-PALL implementation to promote interprofessional exchanges and decision making between nurses and physicians, which was sometimes difficult in this study. Numerous studies support the clinical value of physician–nurse collaboration.^58–61^ An important barrier is when different views exist regarding the nurses’ role in clinical decision-making.^58,59^ In our focus groups, physicians tended to view nurses as executors rather than partners. More interprofessional education on palliative care and time for formal interprofessional meetings are needed.^60^ In addition, the development of a caring culture between professionals, as proposed by Wei et al.,^61^ should help defining common goals of care for the patients.

### Strengths and limitations

This study was carried out in two internal medicine units that were dedicated to COVID-19 until just before the study, and 166 patients with COVID-19 who were hospitalized may have influenced the results because of the fragility of the patients and the heavy workload for professionals. This may have impacted both ID-PALL completion and the observed prevalence of palliative care needs.

The study was initially designed with an interprofessional approach focused on nurses and physicians, who were required to complete ID-PALL jointly. Unfortunately, the reality of clinical practice, perhaps reinforced by the COVID-19 pandemic, did not make this possible, which limited the use of ID-PALL and its recommendations, and thus its clinical impact.

Since this was a monocenter feasibility study, we cannot generalize these results to other places of care.

## Conclusions

The implementation of ID-PALL in a tertiary hospital setting has shown positive results concerning the acceptability and ease of use of the instrument. The prevalence of palliative care needs in the screened patient population was quite impressive. This study also highlighted important challenges in interprofessional collaboration between physicians and nurses. At a practical level, three conclusions can be drawn (1) every effort should be made to encourage systematic screening of patients admitted with ID-PALL in order not to miss out on patients with palliative care needs; (2) for patients in need of general palliative care, specific training of nurses and physicians is required to fully implement the evidence-based recommendations provided with the instrument; and (3) for patients with specialized palliative care needs, an automatic referral to the palliative care team appears necessary to better answer patients’ and relatives’ needs and to improve support for the professionals on the wards. Automatic referrals might also help in easing the moral distress reported by some nurses with regard to palliative care patients.

More generally, the study shows that the implementation of a screening instrument for palliative care needs should be accompanied by specific training and measures fostering interprofessional collaboration to optimize its impact on patient care. Studies on the use of ID-PALL in different clinical contexts (geriatrics and oncology) are currently underway.

## Notes

### Abbreviations Used

CHF: Congestive heart failure
COPD: Chronic obstructive pulmonary disease
GP: General practitioner
ID-PALL: IDentification of patient in need of PALLiative care
PC: Palliative care
SD: standard deviation
SPICT: Supportive and Palliative Care Indicators Tool
