# Supplementary material for: Implementation of the ID-PALL Assessment Tool for Palliative Care Needs: A Feasibility and Prevalence Study in a Tertiary Hospital
Source: Palliat Med Rep. 2024 Aug 5;5(1):350–8. doi: 10.1089/pmr.2023.0080 (PMC11319861; doi:10.1089/pmr.2023.0080)
Supplement: Supplementary Data S1 [file pmr.2023.0080_supplementarydatas1.pdf]

Supplementary material

ID-PALL is freely available in four languages (English, French, Italian, and German) at

www.chuv.ch/id-pall

ID-PALL© G

IDentification of patients in need of General PALLiative Care.
General palliative care is provided by professionals without specialised palliative care training in all care settings and contexts.
Please respond to all of the statements below relative to the patient's current situation:

Space reserved for patient's ID label

1. Would you be surprised if this patient died in the next 12 months?	☐Yes ☐No
2. The patient has a progressive illness or group of illnesses or comorbidities that limits their life expectancy<br>AND presents (select all that are applicable):	
a decline in general functioning (with limited reversibility and an increase in need for support in day to day activities)<br>OR	☐Yes ☐No
a pronounced instability over the last 6 months (defined by: one uncontrolled symptom from the patient's point of view OR a pressure ulcer category ≥3 OR more than one acute delirium episode, infection, unscheduled hospitalisation or fall)<br>OR	☐Yes ☐No
psychosocial or existential suffering (of the patient or people close to them)<br>OR	☐Yes ☐No
the need for support in making decisions during the final stages of life	☐Yes ☐No
3. Current or planned interruption of treatments with curative intent or vital support measures (eg: artificial ventilation, dialysis, artificial feeding or hydration)	☐Yes ☐No
4. Request for comfort care or palliative care from the patient, people close to them or health professionals	☐Yes ☐No

If you have ticked
NO to question 1 OR YES to at least ONE of the statements 2, 3, or 4,
the patient is likely to require general palliative care.
Please complete the ID-PALL S questionnaire on the next page and refer to the general palliative care practice recommendations.

ID-PALL© S

CHUV Palliative and Supportive Care Service

IDentification of patients in need of Specialist PALLiative Care.
Specialised palliative care is provided by or with professionals specialized in palliative care.
Please respond to all of the statements below, relative to the patient's current situation, only when the response to the ID-PALL G is positive:

1. Presence of at least one severe and persistent symptom, including pain, that has not responded satisfactorily to treatment within 48 hours.	☐Yes ☐No
2. Difficulties in evaluating physical symptoms or psychological, social difficulties or spiritual distress	☐Yes ☐No
3. Disagreement or uncertainty on the part of the patient, people close to them or health professionals regarding, for example, medical treatments, resuscitation code or complex decisions	☐Yes ☐No
4. The patient has severe psychosocial or existential suffering (eg: marked symptoms of anxiety or depression, feelings of isolation or of being a burden, loss of meaning or hope, desire to die, or has made a request for assisted suicide)	☐Yes ☐No
5. People close to the patient experience severe psychosocial or existential suffering (eg: marked symptoms of anxiety or depression, major feelings of exhaustion, major disruption to the functioning of the family system, loss of meaning or hope)	☐Yes ☐No
6. Palliative sedation is envisaged (to relieve an intolerable refractory symptom by decreasing the level of consciousness using specific medication)	☐Yes ☐No
7. Advance care plan or advance directives are difficult to establish with the patient and/or people close to them	☐Yes ☐No
8. In your opinion, the patient, people close to them or health professionals could benefit from the intervention of palliative care specialists.	☐Yes ☐No

If you have ticked
YES to ONE of the above statements, the patient is likely to require consultation of a specialist palliative care team.

ID-PALL© v1, 2020, F. Teike Lüthi et al.

ID-PALL-engl

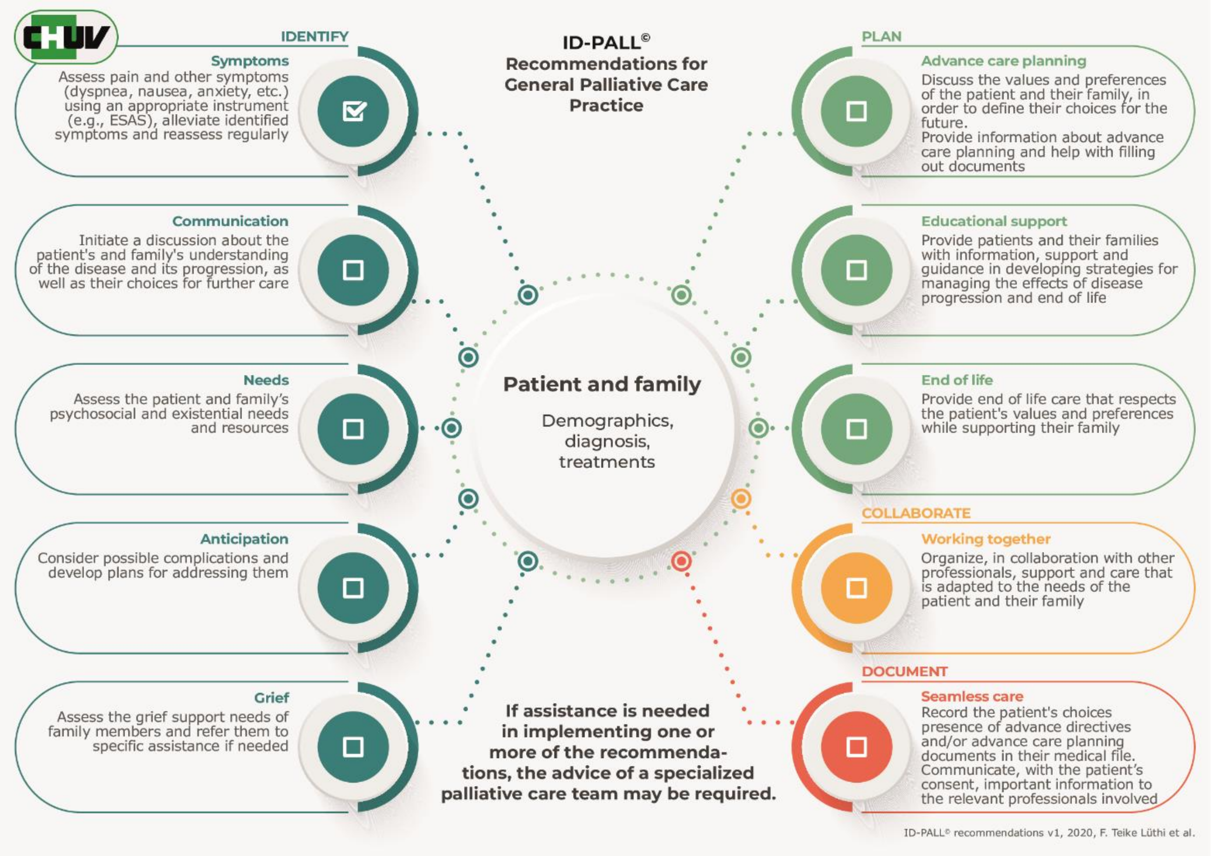

Recommendations-engl
